# Oxaliplatin-related interstitial pneumonia with high-grade fever and relative bradycardia as the presenting signs: a case report

**DOI:** 10.1186/s13256-021-02769-7

**Published:** 2021-04-08

**Authors:** Yasuyuki Taooka, Hiroki Yoke, Junya Inata

**Affiliations:** Department of Respiratory Medicine, Medical Corporation JR Hiroshima Hospital, 3-1-36, Futabano-sato, Higashi-ku, Hiroshima, 732-0057 Japan

**Keywords:** Interstitial pneumonia, Oxaliplatin, Relative bradycardia

## Abstract

**Background:**

Although drug-induced interstitial pneumonia is a well-known adverse side-effect of cancer chemotherapy, the disease is difficult to detect in the early phase. We report a case of oxaliplatin-induced interstitial pneumonia in which eosinophilia and high-grade fever with relative bradycardia were useful presenting signs for the early diagnosis.

**Case presentation:**

A 76-year-old Japanese woman with postoperative recurrent rectal cancer (peritoneal dissemination and liver metastasis) was admitted to our hospital because of productive cough and consolidation on thoracic computed tomography (CT) images. Two months prior to the consultation, she had started chemotherapy (fluorouracil, oxaliplatin, and bevacizumab). After finishing three courses of chemotherapy, she developed fever and was noted to have relative bradycardia. After another two courses of chemotherapy, she developed productive cough, chest discomfort, and high-grade fever. At this time, thoracic CT revealed patchy areas of consolidation distributed predominantly in the periphery. Despite the administration of tazobacterium/piperacillin, the consolidation seen on CT scans gradually worsened. Fiberoptic bronchoscopy was performed, and bronchoalveolar lavage fluid analysis showed increased lymphocytes, eosinophils, and total cell count but a low CD4/ CD8 ratio. No specific pathogen was identified. With a diagnosis of interstitial pneumonia, prednisolone was started and chemotherapy was temporarily discontinued. Her productive cough gradually decreased, and the infiltrative shadows on the thoracic CT scans improved.

**Conclusion:**

Although cases of oxaliplatin-related pneumonia with complicating relative bradycardia are not uncommon, drug-induced interstitial pneumonia should be taken into account in the differential diagnosis. In this case, an increased circulating eosinophil count and high-grade fever with relative bradycardia were the first signs of drug-induced interstitial pneumonia.

## Background

Drug-induced interstitial pneumonia is well known as a major adverse effect of chemotherapy [1], but its detection in the early phase remains difficult. Here we report the case of a patient with rectal cancer who developed oxaliplatin-related interstitial pneumonia. Oxaliplatin is currently used for the treatment of advanced rectal cancer [2]. Although drug eruptions, hypersensitivity, and sensory disturbance of the limbs are well-known adverse effects of oxaliplatin [2–5], drug-induced interstitial pneumonia is also possible. In fact, various cases of oxaliplatin-induced interstitial pneumonia have been reported in the past few decades. In the case reported here, eosinophilia and high-grade fever with relative bradycardia were the first signs of interstitial pneumonia; however, these signs are believed to be rare in this context. Brucellosis, typhoid fever, and drug-induced fever are other known causes of relative bradycardia [6], but the relationship between drug-induced interstitial pneumonia and high-grade fever with relative bradycardia remains uncertain. In this report, we focused on interstitial pneumonia and relative bradycardia.

## Case presentation

A 76-year-old Japanese woman who was being treated for rectal cancer consulted our outpatient clinic with 1-week history of productive cough and fever. She was a never smoker. Two years earlier, she had undergone rectal cancer resection (moderately differentiated tubular adenocarcinoma, stage IIB, T4aN0M0). Two months prior to the consultation, she started chemotherapy with FOLFOX6 (fluorouracil [5-FU], oxaliplatin) + bevacizumab for peritoneal dissemination and liver metastasis from the rectal cancer. After the third course of FOLFOX6 with bevacizumab therapy, she noted fever as high as 39.0 °C without any other symptoms. Physical examination at that time showed a body temperature of 39.1 °C, blood pressure of 138/82 mmHg, heart rate of 85 bpm, respiratory rate of 20 breaths per minute, and intact level of consciousness.  The totally implantable central venous port was palpable on the subclavicular region, without skin inflammation. Although her eosinophil count had increased form 171 to 607/µL, it rapidly decreased to 324/µL 3 days later. Her high-grade fever resolved within a few days without any medications. She then underwent her fourth course of chemotherapy with FOLFOX6 + bevacizumab; the high-grade fever recurred but resolved spontaneously within a few days.

**Fig. 1:**
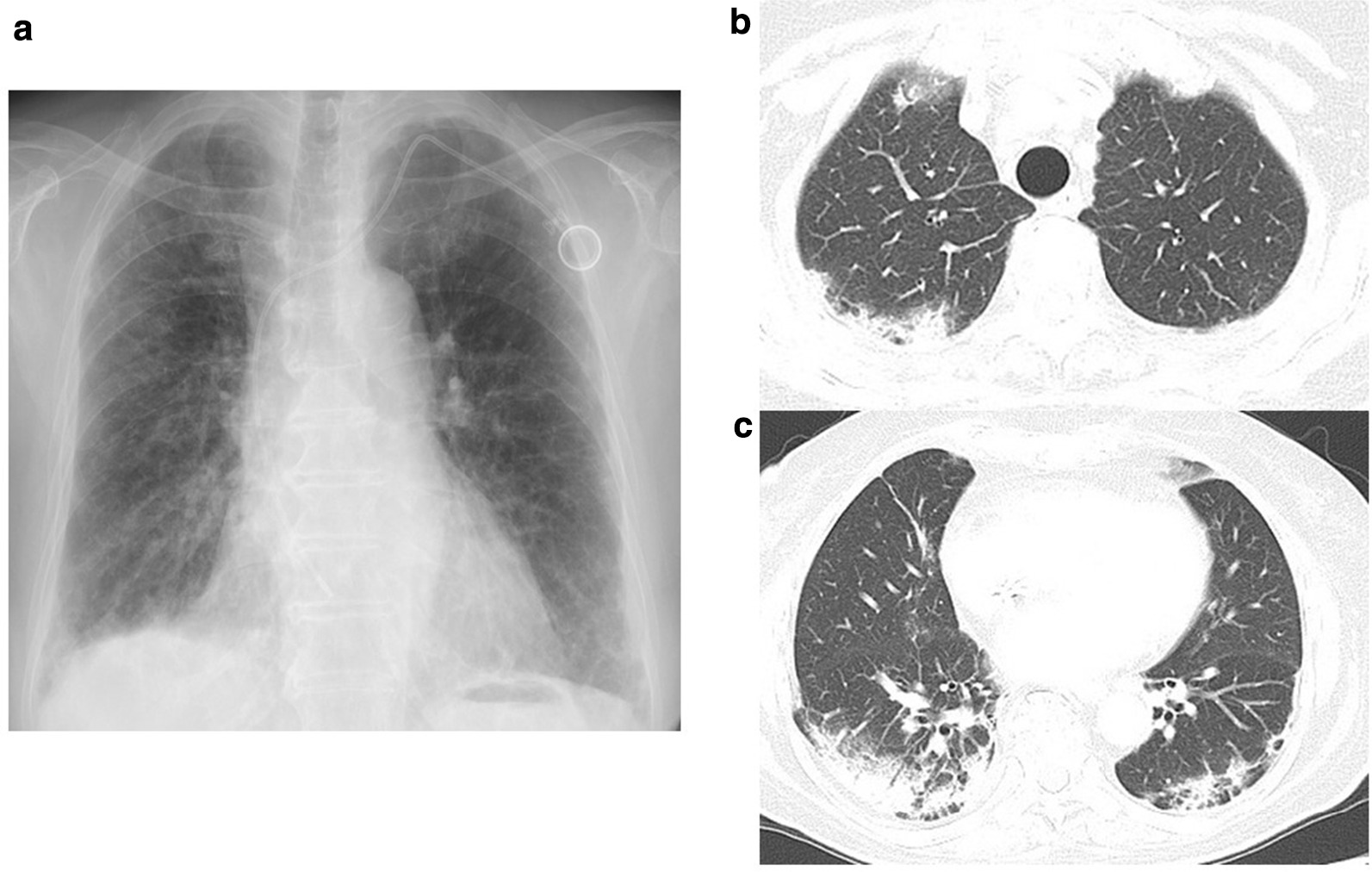
Chest x-ray and thoracic computed tomography (CT) on admission. **a** Chest x-ray shows bilateral, multiple-nonsegmental consolidation. **b**, **c** Thoracic CT performed at the same time shows areas of consolidation, predominantly in the subpleural areas.

**Fig. 2:**
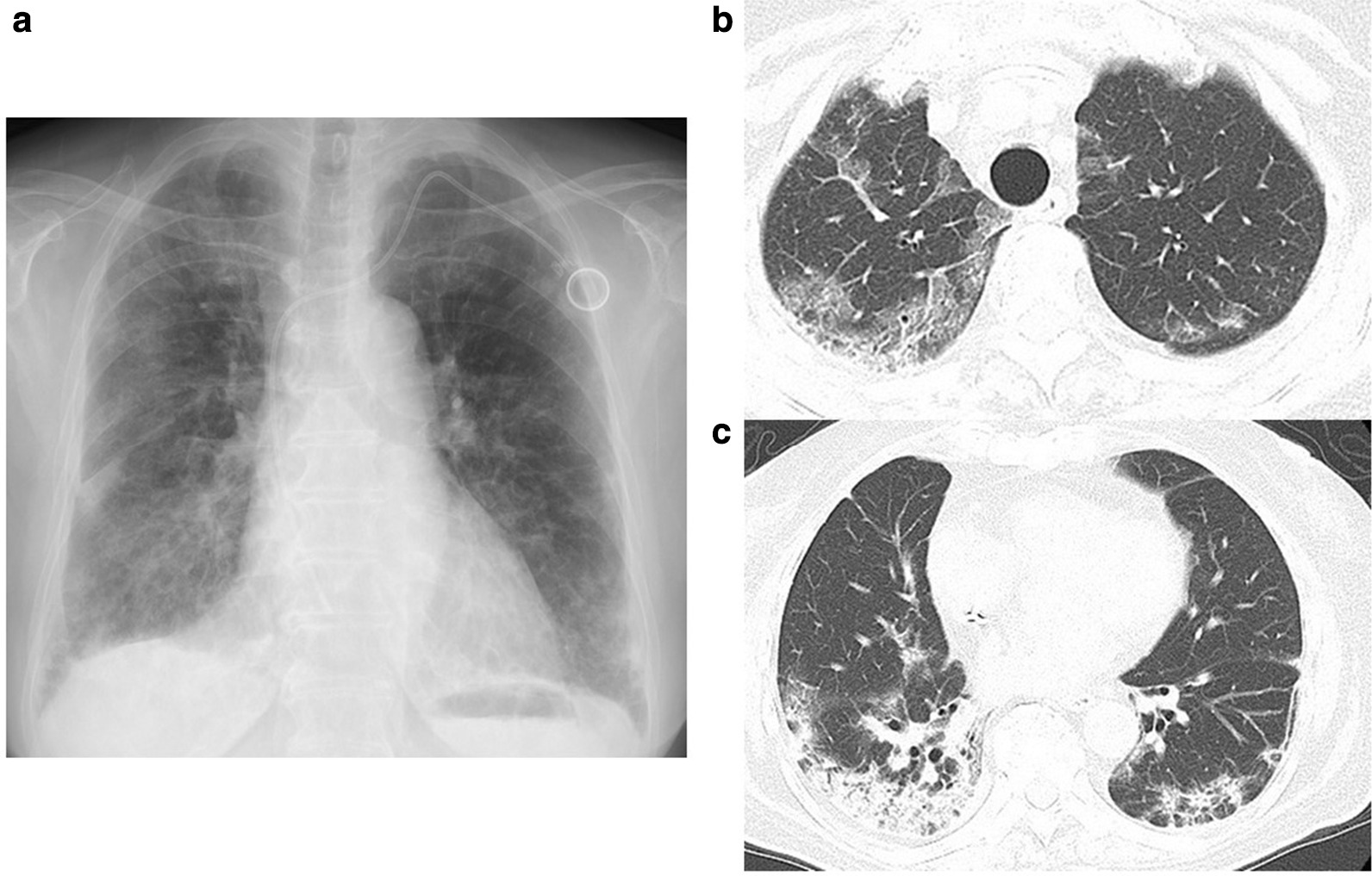
Chest x-ray and thoracic computed tomography (CT) before corticosteroid treatment. Chest x-ray (**a**) and thoracic CT scans (**b**, **c**) show worsening consolidation

After careful observation, the fifth course of chemotherapy was administered; there was recurrence of high-grade fever, along with productive cough and chest discomfort. These symptoms persisted. Physical examination at this time revealed coarse inspiratory crackles on the back. Blood tests revealed a white blood cell count of 5660/µL, eosinophil count of 736/µL hemoglobin of 12.5 g/dL, platelet count of 288,000/mL, and C-reactive protein of 9.92 mg/dL. The laboratory data showed liver dysfunction (aspartate transaminase [AST] 15 IU/L, dysfunctionalanine transaminase [ALT] 19 IU/L, lactate dehydrogenase [LDH] 236 IU/L, alkaline phosphatase [ALP] 401 IU/L, and gamma-glutamyl transpeptidase [γ-GTP] 21 IU/L). Serum surfactant protein-D (SP-D) was 250 U/L and KL-6 was 538 U/L (normal range: 0–499 U/mL). Urine antigen tests for Legionella and *Streptococcus pneumonia* were negative. Bilateral subpleural-predominant areas of consolidation were visible on chest x-ray and thoracic computed tomography (CT) images (Fig. 1). At this point, we highly suspected bacterial pneumonia, rather than interstitial pneumonia, and tazobacterium/piperacillin (TAZ/PIPC) (12.5 g per day) for 5 days was administered. However, her symptoms and the findings on the chest x-ray and CT images gradually worsened (Fig. 2). On physical examination, auscultation of the chest showed early inspiratory coarse crackles. Blood laboratory tests showed a white blood cell count 8960/µL, eosinophil count of 8.8% (788/µL), red blood cell count of 3,310,000/µL, hemoglobin of 10.0 g/dL, hematocrit of 30.8%, platelet count of 349,000/µL, AST of 13 IU/mL, ALT of 10 IU/mL, LDH of 353 U/mL (normal range 106–211 U/mL), C-reactive protein of 4.2 mg/dL, carcino-embryonic antigen (CEA) of 5.5 ng/mL; SP-D was 287 U/L and KL-6 was 1409 U/L.

To rule out interstitial pneumonia and opportunistic infections, fiberoptic bronchoscopy was performed. Bronchoalveolar lavage (BAL) fluid analysis showed lymphocytes at 17.0%, eosinophils at 4.0%, neutrophils at 5.0%, alveolar macrophages at 74.0%, and total cell count of 1.90 × 10^5^/mL. The CD4/CD8 ratio of the BAL fluid was 1.6 (normal range 2.0–3.0). PCR for *Pneumocystis jirovecii* was negative, and no specific pathogen was identified in the BAL fluid. Cytomegalovirus antigenemia was absent. Cell pathological examination of BAL cells showed no evidence of malignancy.

**Fig. 3:**
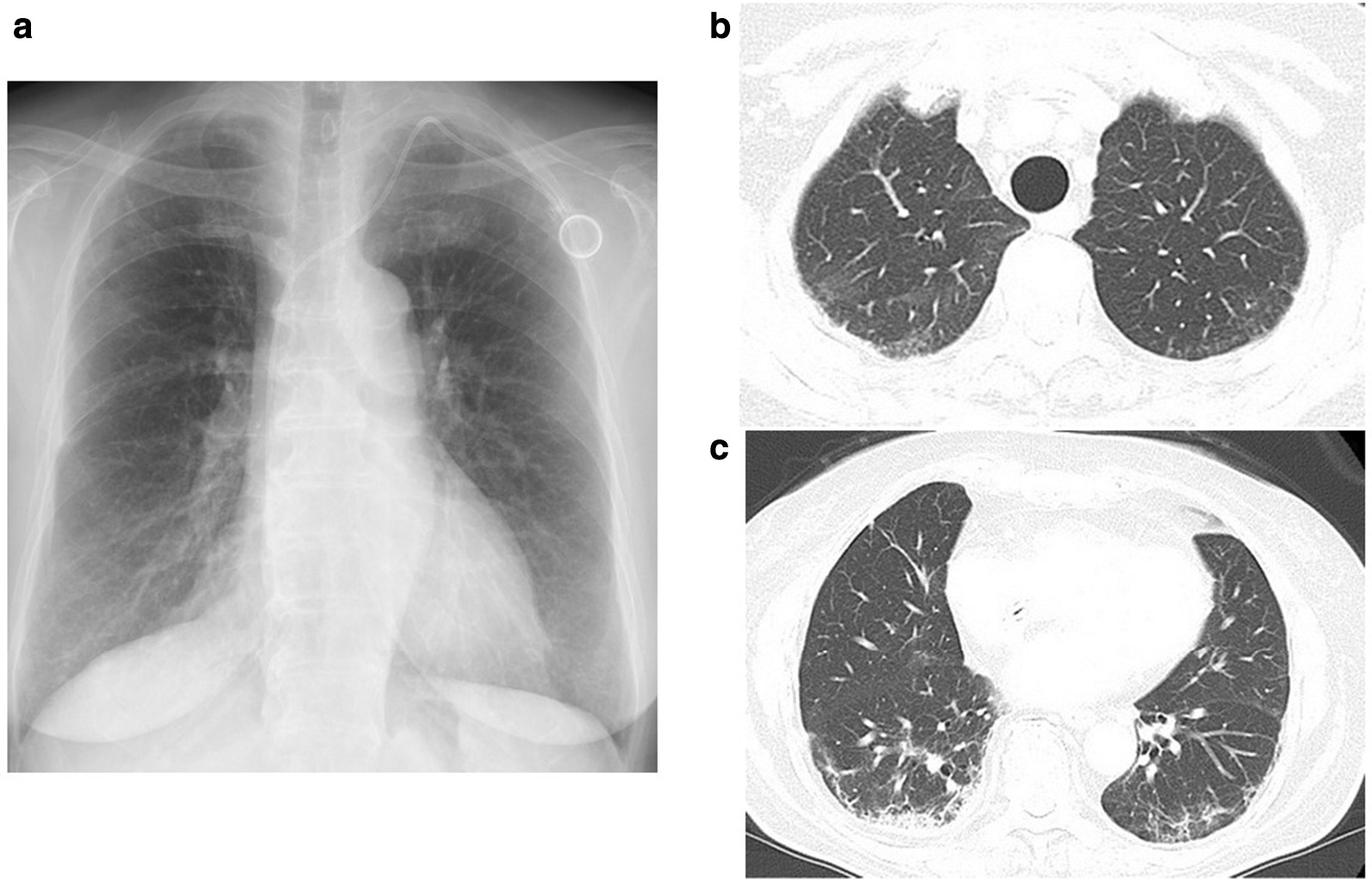
Chest x-ray and thoracic CT after corticosteroid treatment. Chest x-ray (**a**) and thoracic CT scans (**b**, **c**) show improvement of consolidation

**Fig. 4:**
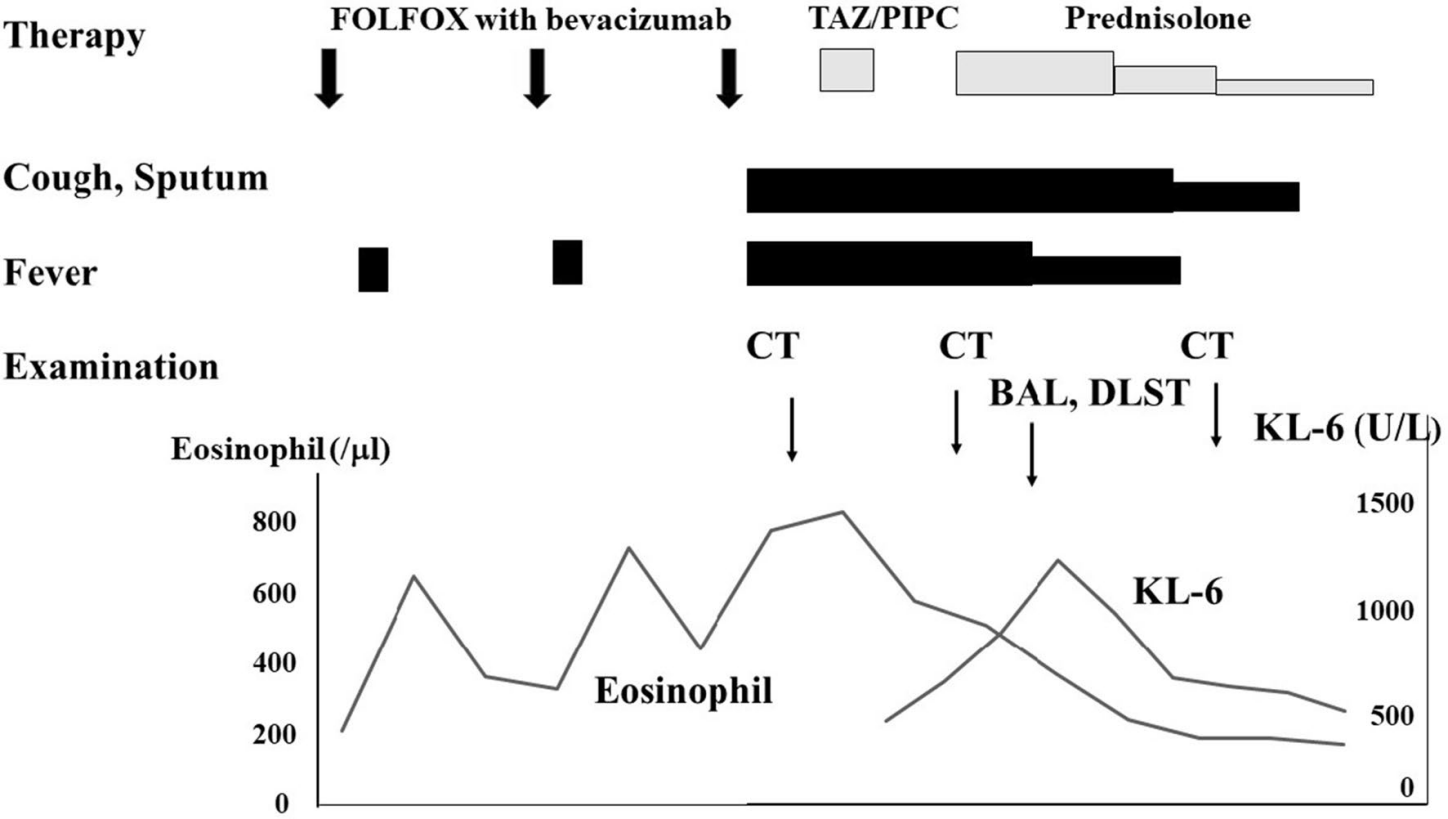
Clinical course of the presented case.* BAL* bronchoalveolar lavage,* DLST* drug-induced lymphocyte stimulation test,* SP-D* surfactant protein-D,* TAZ/PIPC* tazobacterium/piperacillin

A clinical diagnosis of interstitial pneumonia as an adverse effect to chemotherapy was then suspected. The drug-induced lymphocyte stimulation test (DLST) was positive for oxaliplatin but negative for 5-FU and bevacizumab. Because the chest CT findings were similar to those of nonspecific interstitial pneumonia (NSIP) and cryptogenic organizing pneumonia/bronchiolitis obliterans with organizing pneumonia (COP/BOOP), prednisolone at 30 mg daily (0.5 mg/kg/day) was started, and chemotherapy was temporarily discontinued. Her productive cough gradually decreased, and the consolidation seen on chest x-ray and thoracic CT scans improved gradually (Fig. 3). Moreover, the eosinophilia and increased serum SP-D and KL-6 improved. The clinical course of the presenting case is shown on Fig. 4. After 3 months of treatment with tapering doses of prednisolone, there was no recurrence of interstitial pneumonia.

## Discussion and conclusion

The specific feature of the present case was the presence of eosinophilia and relative bradycardia as the first signs of interstitial pneumonia. In this case, because the relationship between drug-induced pneumonitis and relative bradycardia is uncertain, we did not have sufficient cause to discontinue chemotherapy before the diagnosis of interstitial pneumonia was ascertained. Notably, we continued the patient‘s chemotherapy because her high-grade fever after the third course of chemotherapy resolved spontaneously within a few days. On hindsight, that time may have been the optimal point to discontinue chemotherapy. Because the pneumonia was associated with relative bradycardia, intracellular infections, such Legionella pneumonia, could be excluded in this case [6]. To the best of our knowledge, only a few reports have previously described relative bradycardia in oxaliplatin-induced interstitial pneumonia [3, 4]. Based on the patient’s medical history, and confirmed by the patient, there was no medication or medical condition affecting her heart rate. Therefore, we speculated that relative bradycardia might be relevant to the presenting signs.

As we could not prove that oxaliplatin was the only drug that could have induced interstitial pneumonia in its strict definition, we diagnosed as oxaliplatin-related interstitial pneumonia—not oxaliplatin-induced interstitial pneumonia. For this case, we did not try a challenge test. DLST is only one of the ancillary tools and does not confirm the diagnosis of drug-induce pneumonitis. However, if only one drug is positive for DLST, such as in this case, the possibility of that drug being the cause is more likely. For this reason, we found the DLST to be a useful tool in the present case.

 Several cases of oxaliplatin-induced interstitial pneumonia have been reported to have shown a NSIP-like pattern [2–5]. Although results from the BAL fluid analysis and the CT pattern of drug-induced pneumonitis vary widely, the findings in this case were consistent with those reported in these earlier cases. Moreover, the patient’s response to corticosteroid therapy was useful in supporting our speculation. Because of temporary hypoxia during bronchoscopy, transbronchial lung biopsy was not performed in this patient; therefore, we could not differentiate between NSIP and COP/BOOP. Moreover, the pathological finding could not rule out the possibility of drug-induced pneumonitis. Nevertheless, this did not affect the choice of treatment for this case. Chronic eosinophilic pneumonia was considered in the differential diagnosis, based on the CT findings, but it was ruled out based on the results of BAL fluid analysis.

In summary, we report a case of oxaliplatin-related interstitial pneumonia that was successfully treated with corticosteroid. When a finding of nonresolving pneumonia is seen on chest x-ray during chemotherapy for rectal cancer, the possibility of an adverse effect to oxaliplatin should be considered. Nonsegmental and multiple areas of consolidation distributed predominantly in the periphery were the characteristic findings in the present case. Moreover, an increased circulating eosinophil count and high-grade fever with relative bradycardia may be useful for the early diagnosis of this condition.

## Data Availability

Not applicable

## Abbreviations

CT: Computed tomography
BAL: Bronchoalveolar lavage
DLST: Drug-induced lymphocyte stimulation test
NSIP: Nonspecific interstitial pneumonia
COP/BOOP: Cryptogenic organizing pneumonia/ bronchiolitis obliterans with organizing pneumonia
SP-D: Surfactant protein-D
TAZ/PIPC: Tazobacterium/piperacillin
